# Within and Beyond the Nucleotide Addition Cycle of Viral RNA-dependent RNA Polymerases

**DOI:** 10.3389/fmolb.2021.822218

**Published:** 2022-01-10

**Authors:** Peng Gong

**Affiliations:** ^1^ Key Laboratory of Special Pathogens and Biosafety, Wuhan Institute of Virology, Center for Biosafety Mega-Science, Chinese Academy of Sciences, Wuhan, China; ^2^ Drug Discovery Center for Infectious Diseases, Nankai University, Tianjin, China

**Keywords:** RNA virus, RNA-dependent RNA polymerase, nucleotide addition cycle, translocation, cleavage, misincorporation, nucleotide analog

## Abstract

Nucleotide addition cycle (NAC) is a fundamental process utilized by nucleic acid polymerases when carrying out nucleic acid biosynthesis. An induced-fit mechanism is usually taken by these polymerases upon NTP/dNTP substrate binding, leading to active site closure and formation of a phosphodiester bond. In viral RNA-dependent RNA polymerases, the post-chemistry translocation is stringently controlled by a structurally conserved motif, resulting in asymmetric movement of the template-product duplex. This perspective focuses on viral RdRP NAC and related mechanisms that have not been structurally clarified to date. Firstly, RdRP movement along the template strand in the absence of catalytic events may be relevant to catalytic complex dissociation or proofreading. Secondly, pyrophosphate or non-cognate NTP-mediated cleavage of the product strand 3′-nucleotide can also play a role in reactivating paused or arrested catalytic complexes. Furthermore, non-cognate NTP substrates, including NTP analog inhibitors, can not only alter NAC when being misincorporated, but also impact on subsequent NACs. Complications and challenges related to these topics are also discussed.

## Introduction

RNA viruses are a large collection of diverse, rapidly evolving viruses with a wide host range covering bacteria and eukaryotes (Krupovic et al., 2018). In recent years, emerging and re-remerging RNA viruses causing human and animal diseases have posed a great impact to our daily life. Understanding the fundamental features of RNA viruses has become an attractive and rapid growing research area ever since the emergence of severe and acute syndrome coronavirus 2 (SARS-CoV-2) causing the coronavirus disease 2019 (COVID-19) (Zhou et al., 2020). Effective antivirals and vaccines are in urgent need for prevention and control of known and future RNA virus pathogens. One unique feature of RNA viruses is that their genome replication and transcription processes are DNA-independent, thus requiring a virally-encoded RNA-dependent RNA polymerase (RdRP) to carry out these essential processes of the virus life cycle (Wolf et al., 2018). Due to their essentialness and highest conservation level, RdRPs have become attractive targets to develop antivirals with high potency and/or broad-spectrum potential. Although being considered the most conserved protein of RNA viruses, RdRPs are still quite diverse with respect to their global structure organization (Lesburg et al., 1999; Thompson and Peersen, 2004; Lu and Gong, 2013; Pflug et al., 2014; Liang et al., 2015; Jia and Gong, 2019; Kirchdoerfer and Ward, 2019), initiation mechanisms (Butcher et al., 2001; Reich et al., 2014; Appleby et al., 2015; Zhang et al., 2021), and regulation by host and viral factors (Kidmose et al., 2010; Liu et al., 2018; Chen et al., 2020; Yan et al., 2021a). Nucleotide addition cycle (NAC), a process shared by all nucleic acid polymerases carrying out NTP/dNTP-driven phosphodiester bond formation (Huang et al., 1998; Li et al., 1998; Yin and Steitz, 2004; Kornberg, 2007; Gong and Peersen, 2010), may thus be the most conservative part of viral RdRP working mechanisms. Understandings of common features in RdRP NAC can help describe the viral genome replication and transcription processes that are composed of thousands of NACs and can benefit development of nucleot(s)ide analog drugs targeting viral RdRPs (Gong, 2021; Johnson and Dangerfield, 2021).

**FIGURE 1:**
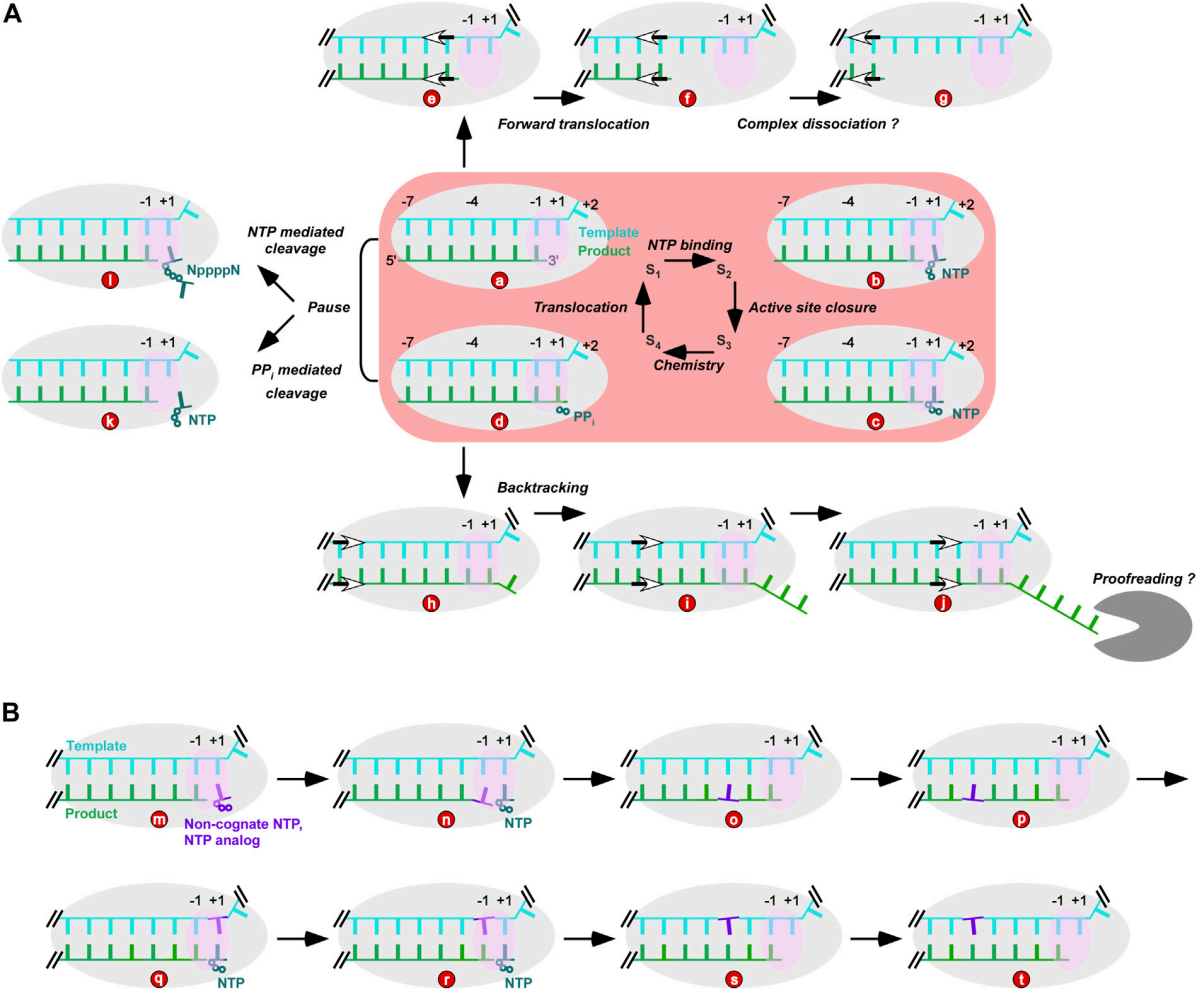
Viral RdRP NAC and possibly related processes. **(A)** RdRP NAC and its relationship with nucleotide addition-free translocation events and intrinsic product cleavage activities. (a–d): four reference states of NAC; e–g: states derived from RdRP forward translocation; (h–j): states derived from polymerase backtracking; d to k: PP_i_ mediated product cleavage; d to l: non-cognate NTP-mediated cleavage. The gray “pacman” represents a virally encoded exonuclease. **(B)** NAC intervention by incorporation of non-cognate NTP or NTP analogs. Gray and pink ovals represent RdRP and its active site, respectively. Wherever necessary, double slashes were used to indicate continuation of an RNA strand.

NAC of nucleic acid polymerase typically contain four microsteps: NTP/dNTP binding, active site closure, phosphoryl transfer reaction (chemistry), and translocation, while four structural reference states (S_1_-S_4_) are usually used to help depict these microsteps (Figure 1A, central part with pink background). Critical conformational changes have been found accompanying active site closure (S_2_ to S_3_) and translocation (S_4_ to S_1_) (Temiakov et al., 2004; Yin and Steitz, 2004; Wang et al., 2006). In viral RdRPs, a unique palm-domain-based conformational change takes place upon active site closure (Zamyatkin et al., 2008; Gong and Peersen, 2010; Appleby et al., 2015). By contrast, A-family polymerases represented by bacteriophage T7 RNA polymerase and *Thermus aquaticus* (Taq) DNA polymerase close the active site through a large-scale conformational change of their fingers domain (Li et al., 1998; Yin and Steitz, 2004). S_3_ and S_4_ structures that both have a closed active site have been solved in multiple polymerase systems, and consistently demonstrate the critical role of two divalent metal ions in forming the transition state of the phosphoryl transfer reaction (Steitz and Steitz, 1993). Two types of RdRP translocation intermediate structures both highlight an asymmetric movement of the template-product RNA strands and stringent control of the template RNA movement by the RdRP-specific template-interacting motif G (Shu and Gong, 2016; Wang et al., 2020a). Similar intermediates have been observed with transcriptional pausing-related multi-subunit DNA-dependent RNA polymerases (DdRPs) (Guo et al., 2018; Kang et al., 2018), suggesting that this phenomenon may be shared even by structurally unrelated polymerase families.

Although numerous NAC-state-related structures from various viral RdRPs have provided a relatively complete structural view of the cognate NTP-driven NAC, several aspects directly or indirectly related to NAC have rarely been addressed structurally in RdRPs including nucleotide addition-free translocation, intrinsic product cleavage activities, and perturbation of NAC by non-cognate NTPs and NTP analogs. As discussed below, these events are possibly related to important events including but not limited to RdRP catalytic complex dissociation, proofreading, reactivation, fidelity control, and effective intervention. In the following sections, a synoptic introduction of these events and structural challenges to approach them are discussed.

## RdRP Translocation Beyond NAC

S_1_ and S_4_ represent the post- and pre-translocation states, respectively. In a classic NAC, a post-translocation S_1_ complex directs the binding of the incoming NTP, while the polymerase in the pre-translocation S_4_ complex needs to translocate to the next register and reopen its active site for the next NAC (Figure 1A, central panel with pink background). By contrast, “forward” movement from the post-translocation S_1_ and “backward” movement from the pre-translocation S_4_ of the polymerase can move the polymerase out of an NAC. The former, if occurs successively, makes the 3′-end of the product RNA move toward the upstream. The template-product duplex in contact with the polymerase thus shortens and may lead to dissociation of the complex (Figure 1Aa,e–g). These forward-translocated states occur in DdRPs during intrinsic transcription termination or in halted transcription elongation complexes, both resulting in dissociation of the polymerase complex (Yarnell and Roberts, 1999; Zhou et al., 2007). Similar situations may also be induced by other nucleic acid binding proteins or nucleic acid elements such as bacterial translocase Rho, bacterial transcription-repair coupling factor Mfd, or the class I transcription termination signal in T7 RNA polymerase transcription (Kassavetis and Chamberlin, 1981; Macdonald et al., 1994; Deaconescu et al., 2006; Murphy et al., 2009; Roberts, 2019). However, dissociation routes unrelated to RdRP forward translocation cannot be ruled out. Furthermore, a Brownian ratchet model applied to DdRPs suggests that polymerase can slide along the nucleic acid template driven by thermal motion with the binding of incoming NTP serving as the “ratchet” to favor the forward-translocated state (Guajardo and Sousa, 1997; Vassylyev and Artsimovitch, 2005). Therefore, RdRP may forward translocate simply through “diffusing” along the template even without *cis*-acting elements or *trans*-acting factors.

The “backward” movement of the polymerase, better known as backtracking in DdRP transcription, results in unraveling of the template-product duplex downstream of the +1 site (the position where incoming NTP binds during NAC). In DdRPs, there is a channel to accommodate the single-stranded 3′-portion of the product RNA and transcription factors such as bacterial GreA/GreB and eukaryotic TFIIS can facilitate intrinsic endonuclease activity of DdRP to reactivate transcription (Abdelkareem et al., 2019; Bradley et al., 2019; Wang et al., 2009). Proofreading activities in viral RdRPs have been found in CoVs, with the virally encoded nsp14 protein utilizing its exonuclease module to excise the 3′-nucleotide(s) of the product RNA (Subissi et al., 2014) (Figure 1Ad,h–j). Backtracking-related proofreading models were proposed in SARS-CoV-2 RdRP studies, in which the viral helicase hypothetically facilitates RdRP backtracking upon misincorporation and the 3′-end of the product therefore is delivered to the exonuclease active site for cleavage after multiple rounds of translocation events (Chen et al., 2020; Yan et al., 2021b).

Except for recently reported backtracked SARS-CoV-2 RdRP structures obtained using RNA scaffolds with designed mismatches at the 3′-portion of the product RNA (Malone et al., 2021), structures with the 3′-end of the product strand poise upstream of position −1 (the position of the priming nucleotide of the product strand) or downstream of position +1 have rarely been captured in viral RdRPs. Nevertheless, these nucleotide-addition-free translocation events likely occurs and may indeed play important roles under certain circumstances. On one hand, assembling an RdRP-RNA complex with other factors may help visualize the forward translocated or backtracked states. On the other hand, RdRP variants (i.e., from different viruses or virus strains) or mutants with altered nature in controlling the movement of either the template or the product strand may have different odds in capturing these states by structural biology approaches.

## RdRP Intrinsic Cleavage Activities and Their Relevance to Reactivation

Pyrophosphate (PP_i_) is the byproduct of the NTP/dNTP-driven phosphoryl transfer reaction in NAC. Under certain circumstances (e.g., high PP_i_ concentration in polymerase assays), PP_i_ can reverse the reaction through pyrophosphorolysis (Figure 1Ac,d–k). Pyrophosphorolysis can not only participate in proofreading by excising the non-cognate nucleotide, but also play regulatory roles by modulating the overall progress of nucleic acid synthesis (Imashimizu et al., 2019). While PP_i_ can be observed in pre-translocation polymerase complex structures (Yin and Steitz, 2004; Gong and Peersen, 2010), it has not been observed in polymerase structures that has completed translocation, suggesting that PP_i_ release and translocation likely coincide in timing. Starting from a post-translocation S_1_ complex crystal, an interesting reverse translocation intermediate structure was captured in an enterovirus RdRP with PP_i_ present in the soaking solution (Wang et al., 2020a). In such a structure, the 3′-nucleotide of the product moved from position −1 to almost position +1. Although pyrophosphorolysis did not occur in the crystal, solution trials mimicking the crystal soaking condition led to observation of PP_i_-mediated cleavage (Wang et al., 2020a) (Figure 1Ad–k), suggesting reverse translocation as a prerequisite of the cleavage of a post-translocation complex. Another interesting observation in this structure is the “slippage” between the template-product RNA, resulting in a duplex only partially matched. Completion of this reverse translocation thus requires realigning of the two strands. Such a slippage-and-realigning process was not observed in regular forward translocation, in which basepairing interactions between the two strands were maintained (Shu and Gong, 2016; Peersen, 2017; Wang et al., 2020a). Hence, the reverse translocation is relatively energetically-unfavorable.

Another known polymerase activity cleaving the 3′-nucleotide of the product strand is mediated by non-cognate NTPs. First reported in human immunodeficiency virus 1 (HIV-1) reverse transcriptase (RT), a non-cognate NTP can induce the cleavage of the product strand 3′-nucleotide, forming a dinucleotide with a 5–5′ poly-phosphate (tetra- or tri-phosphate) linkage when the incoming cognate dNTP is not available (Meyer et al., 1998). Similar activities were subsequently observed in hepatitis C virus (HCV) RdRP (Jin et al., 2013a). In both systems, a chain terminating nucleotide can be cleaved by this NTP-mediated activity to yield an extendable 3′-end, and thus implying its potential role in proofreading.

Unlike CoVs, many other RNA viruses do not encode an exonulease and thus sometimes are considered error-prone. However, PP_i_- and NTP-mediated cleavage activities by RdRP itself may play key roles in maintaining viral genome stability and keeping the virus away from the error-catastrophe threshold (Crotty et al., 2001). Furthermore, RdRP pausing or arrest caused by RNA elements or regulatory proteins can in principle also be resolved by these activities. As polymerases can pause at either pre- or post-translocation NAC states (Figures 1Aa,d) but both cleavage activities likely occur at pre-translocation state (Figure 1Ad–l), polymerase backtracking to the pre-translocational position for cleavage may increase the opportunity of rescuing the complex from a “trapped” status. To date, the structural basis of the NTP-mediated cleavage has remained elusive, while structural understandings of pyrophosphorolysis can be readily achieved through its reverse steps of nucleotide addition and the preceding reverse translocation.

## RdRP NAC Regulation by Non-cognate NTP or NTP Analogs

As the major source of nucleotide mutations, misincorporation by nucleic acid polymerases and its mechanisms are of great interest in understanding evolution of species. Misincorporations, often considering basepairing mismatch derived events, occur at a rate of 10^−3^–10^−5^ if not considering proofreading (Drake and Holland, 1999; Chen et al., 2000; Johnson et al., 2000; Zhang et al., 2000). Therefore, it is generally difficult to capture misincorporation-related polymerase structural states. A classic work in this aspect is from a systematic study in *Bacillus stearothermophilus* DNA polymerase I fragment (BF), a high-fidelity DNA polymerase (Johnson and Beese, 2004). By attempting every possible mismatched base pair combination, multiple mismatch-containing crystal structures were solved at atomic resolution, depicting various types of mismatches and their direct impact on NAC. By solving a set of structures with extension of a G:T mismatch in successive NACs, distortion of a mismatch up to six register from the 3′-end of the product was found to have an impact on the active site through long-range transmission. By contrast, systematic structure determination of mismatch-containing RdRP catalytic complexes is lacking.

The successful usage (Rubin et al., 2020) of nucleotide/nucleoside analog (NA) drugs in treating RNA virus related disease have emphasized the importance of this class of compounds in prevention and control of existing and future pathogens (Furuta et al., 2013; Gane et al., 2013; Rubin et al., 2020). However, multiple factors including but not limited to the differences among RdRP active sites, differences in optimal prodrug forms targeting certain cell types, and emergence of drug-resistant virus strains determine the effectiveness of an NA on a certain virus and its broad-spectrum potential (Feng and Ray, 2021; Jia et al., 2021; Seley-Radtke et al., 2021). Understanding the intervention mechanism of the NTP form of NA (the effective molecule *in vivo*) at enzymology and structural levels is a key not only to identify repurposed NA drugs, but also to design new NA drugs (Appleby et al., 2015; Xu et al., 2017). As an example, remdesivir (RDV), a ribose 1′-substituted adenosine analog, was first developed for Ebola treatment and was found effective on other viruses including various CoVs (Cho et al., 2012; Jacobs et al., 2016; Agostini et al., 2018; Gordon et al., 2020a). A delayed intervention by the NTP form of RDV (RDV-TP) likely at the third NAC after the first incorporation was found in CoVs and subsequently in enterovirus 71 (EV71) (Gordon et al., 2020b; Wu et al., 2021), demonstrating both uniqueness and diversity of this compound in RdRP intervention. Further characterizations revealed that structurally equivalent S861 and S417 in SARS-CoV-2 and EV71 RdRPs are responsible for this delayed intervention likely through steric hindrance of the RDV 1′-cyano group, and under certain circumstances the incorporated RDV (i.e., the monophosphate form of RDV) can overcome this serine roadblock (Tchesnokov et al., 2020; Kokic et al., 2021; Seifert et al., 2021; Wu et al., 2021). The non-terminating feature of RDV intervention awaits further investigation considering the entire replication/transcription process as well as viral protein translation if RDV-containing transcripts are utilized to direct protein synthesis. The 1′-substitutions are therefore endowed with broad-spectrum and delayed intervention potentials in NA drug development. Another notable NA type is represented by ribavirin, favipiravir, and molnupiravir, all with ambiguous basepairing capability (Crotty et al., 2000; Jin et al., 2013b; Gordon et al., 2021). For example, the N4-hydroxyl cytosine of molnupiravir was structurally captured to direct the incorporation of either a GMP or an AMP in SARS-CoV-2 RdRP (Kabinger et al., 2021). Unlike immediate chain-terminating NAs or RDV, these mutagenic NAs likely generate antiviral effects by driving the viral population beyond the error-catastrophe threshold.

To date, structures of RdRP-RNA complexes containing non-cognate NTP-derived mismatches are rarely reported, while related structures depicting intervention mechanisms of representative NAs have been emerging with the fast growing of RNA virus research (Ferrer-Orta et al., 2007; Appleby et al., 2015; Wang et al., 2020b; Kabinger et al., 2021; Kokic et al., 2021). NAs with immediate chain terminating features may directly interfere with the NAC at the +1 or -1 site (Figure 1Aa–d,Bm–n); those are not immediately terminating can possibly propagate its impact on NAC from remote sites (Figure 1Bo,p); incorporated NAs that eventually become part of the full-length product may affect NAC when reaching the active site and the template-product binding regions as a template nucleotide (Figure 1Bq–t). Together with enzymology characterizations, solving more NA-containing RdRP-RNA structures with representative modifications and intervention mechanisms will provide key references for cell- and animal model-based NA effectiveness assessment and a comprehensive pre-clinical evaluation of NA drug candidates.

## Discussion

Representative NAC-related RdRP-RNA complex structures have been reported in picornaviruses, HCV, influenza viruses, bacteriophage ϕ6, and more recently in bunyaviruses and CoVs (Butcher et al., 2001; Gong and Peersen, 2010; Gong et al., 2013; Appleby et al., 2015; Arragain et al., 2020; Wang et al., 2020b; Wandzik et al., 2020). However, a comprehensive structural understanding of NAC requires much more representative RdRP systems and determination of RdRP structures at different phases of its replication and transcription. In most cases, the replication/transcription complex (RTC) works as a multi-subunit machinery and its components vary in different processes and at different stages of a certain process (Reed and Rice, 2000; Bollati et al., 2010; Smith and Denison, 2013). Therefore, structures building on RdRP-RNA complex with other RTC components in the assembly are also highly valuable and in some cases more functionally relevant. Continuing progress in cryo electron microscopy (cryo-EM)-related techniques and deep learning-based structure prediction of biological macromolecule and its complexes have been providing a boost in understanding the RTC as well as the NAC carried out by it (Frank, 2018; Tunyasuvunakool et al., 2021).

## Data Availability

The original contributions presented in the study are included in the article/supplementary material, further inquiries can be directed to the corresponding author.
